# Unexpected diagnosis of acute lymphoblastic leukemia in a 2-year-old with acute appendicitis – Case report

**DOI:** 10.1016/j.ijscr.2021.106077

**Published:** 2021-06-09

**Authors:** Scott R. Marison, Brooke Pati, Nicole R. Laferriere, Russell K. Woo, Ally Ha

**Affiliations:** a The Queens Medical Center, Department of Surgery, 1356 Lusitana Street, 6th Floor, Honolulu, HI 96813, United States of America; b Tripler Army Medical Center, Department of Surgery, 1 Jarrett White Rd, Honolulu, HI 96859, United States of America; c Kapiolani Medical Center for Women and Children, Department of Pediatric Surgery, 1319 Punahou St, Honolulu, HI 96826, United States of America

**Keywords:** Case report, Leukemia, Appendicitis, Appendectomy, Pathology

## Abstract

**Introduction and importance:**

Appendicitis is an extremely common surgical problem, especially in the pediatric population. However, leukemic infiltration of the appendix is rare and even more so is having acute appendicitis as the initial manifestation.

**Case presentation:**

The patient is a 2-year-old female with multiple febrile illnesses since birth, who presented to the emergency department with a 3-day history of abdominal pain, fever, and decreased appetite. Ultrasound of her right lower quadrant was consistent with acute appendicitis. A laparoscopic appendectomy was performed successfully without complication. However, pathological examination of the specimen revealed an appendix with partial involvement of B-lymphoblastic lymphoma/leukemia in a background of lymphoid hyperplasia. This prompted referral to a pediatric hematologist/oncologist. Further workup revealed abnormal immature cells on peripheral blood flow cytometry. Bone marrow biopsy confirmed a diagnosis of B-cell acute lymphoblastic leukemia.

**Clinical discussion:**

Though acute appendicitis is very common and management is well documented, it is rare for pathological examination to uncover leukemia as an underlying etiology and to have acute appendicitis as the initial manifestation of hematologic malignancy. To our knowledge, very few similar events have occurred and been documented in the medical literature.

**Conclusion:**

Physicians and surgeons should be aware that, though quite rare, leukemic infiltration of the appendix can occur and should be considered in the differential diagnosis of acute appendicitis. Notably, pathologic examination of the appendix may be particularly informative. Diligent follow-up of abnormal pathology is crucial in cases suggestive of underlying hematologic malignancy.

## Highlights

• Acute appendicitis is a rare initial manifestation of hematologic malignancy.
• Histopathological evaluation of surgical specimens is essential in patient care.
• Atypical presentations of acute appendicitis can lead to a delay in diagnosis.
• Signs of acute appendicitis may be masked in neutropenic patients.

## 1 Introduction

Appendicitis is the most common abdominal emergency requiring surgery with a lifetime risk of about 7% with peak incidence in the late teens and early twenties [1,2]. Acute appendicitis is a known complication of treatment for hematologic malignancies, with a reported incidence of 0.5% to 4.4%, but involvement of the appendix with leukemic cells is rare [[3], [4], [5], [6], [7], [8], [9]]. Even more rare is the identification and diagnosis of ALL as a result of a clinical presentation of acute appendicitis.

ALL is a malignant neoplasm of hematopoietic stem cells of lymphoid lineage arising in the bone marrow, and it is the most common malignancy in childhood. Precursor B-cell neoplasm (B-cell ALL) is primarily seen in early childhood, is without sex bias and accounts for nearly 85% of cases, while T-cell ALL has a male predilection with a peak incidence in preadolescent children. Initial symptoms of ALL are often nonspecific and may be difficult to distinguish from other self-limited diseases. A meta-analysis of over 3000 children from 33 studies reported that 6% of children were asymptomatic at the time of diagnosis [10]. The authors also found that of symptomatic children with ALL, more than half presented with at least one of the following features: palpable liver or spleen, pallor, fever or bruising [10].

Immunohistochemistry typically demonstrates expression of B cell antigens without T cell antigens. These include CD19, cytoplasmic CD 79a, and cytoplasmic CD 22. Sometimes they can also be positive for CD 10, CD 24, PAX5, and TdT. CD 20 and CD 34 are variable and CD45 is usually absent [11]. Mainstay of treatment for ALL is chemotherapy with a reliance on multidrug regimens to avoid development of resistance. The overall survival for this disease process is approximately 80%, with certain subsets experiencing greater than 98% cure rate [12].

This work has been reported in line with the SCARE criteria [13].

## 2 Presentation of case

A 2-year-old female with a history of constipation presented with a 3-day history of acute onset abdominal pain, fever, and decreased appetite. Her mother noted an episode of diarrhea the day prior to presentation but denied any vomiting or recent sick contacts. Further questioning revealed a history of numerous febrile illnesses since birth. She was born full-term after an uncomplicated pregnancy. All vaccinations were up to date and developmental milestones were met.

She was febrile to 101.2 F and hemodynamically normal. Examination revealed a soft and non-distended abdomen with mild diffuse tenderness to palpation. There was no hepatosplenomegaly or lymphadenopathy. Biochemical studies were significant for leukopenia of 4.1 × 10^9^/L and an absolute neutrophil count of 574 with 7% atypical lymphocytes. A right lower quadrant ultrasound demonstrated an 8 mm non-compressible, hyperemic, and tender appendix without signs of free fluid or abscess. A diagnosis of acute appendicitis was established and she was consented for laparoscopic appendectomy, which was performed by the authors. A standard, three port laparoscopic appendectomy was performed with carbon dioxide pneumoperitoneum. The mesoappendix was divided with electrocautery and the appendix was divided at its base using a single fire of a laparoscopic surgical stapler. She tolerated the procedure well and there were no complications. Intraoperative findings were consistent with acute, non-perforated appendicitis. She had an uneventful postoperative course and was discharged home two days after her procedure.

Final pathology of the specimen not only revealed the presence of acute appendicitis with microscopic perforation and acute serositis, but also partial involvement of B-lymphoblastic lymphoma/leukemia in a background of lymphoid hyperplasia. Notably, immunohistochemistry revealed an increase in CD10+ and TdT+ lymphoid cells in perivascular locations and the periphery of the main appendiceal lymphoid tissue. Additional immunohistochemistry showed the CD10+ and TdT+ cells to have uniform coexpression of CD19, bcl-2, and CD43, with minimal HGAL, and no CD3. Other findings included CD20+/PAX5+ B cells noted in the lymphoid follicles, and Bcl-6+ lymphocytes confined to the reactive-appearing germinal centers.

The patient was subsequently referred to pediatric hematology/oncology for evaluation and further workup. History was negative for leg or bone pain, there was no family history of pediatric cancers, and examination revealed no masses, adenopathy, hepatosplenomegaly, or increased bleeding, bruising, petechiae, or pallor. Peripheral blood flow cytometry demonstrated that 31% of cells expressed immature markers concerning for B-cell acute lymphoblastic leukemia versus benign hematogone. She underwent a bone marrow biopsy which confirmed the diagnosis of B-cell acute lymphoblastic leukemia (ALL). The patient was enrolled in the AALL1131 trial and underwent induction therapy, which was complicated by hemorrhagic cystitis, Enterobacter urinary tract infection, and acute renal failure requiring Continuous Renal Replacement Therapy. She recovered from her acute renal failure and is currently on maintenance therapy for B-cell ALL.

## 3 Discussion

There are only four cases in the medical literature documenting tumoral infiltration of the appendix causing acute appendicitis [3,9,14]. One report discussed leukemia found within an appendix removed for appendicitis in a 71-year-old male who presented with right lower quadrant pain [3]. He underwent laparoscopic appendectomy with pathology revealing diffuse infiltration of large atypical cells. Bone marrow biopsy confirmed the diagnosis of acute myelogenous leukemia. He underwent chemotherapy, but unfortunately died from his disease 49 days later.

One study retrospectively looked at the histology of appendectomy specimens to find unusual pathologies [1]. Unusual findings were seen in 1.7% of cases. The most common findings were *Enterobius vermicularis* and carcinoid with lymphoma only occurring in 14 of 1366 cases and Leukemia seen in 4 of 1366 cases. In their review, the incidence of leukemic appendicitis was about 0.005%.

There are studies that discuss cancer risk after appendectomy [2,15]. One cohort study compared those who underwent an appendectomy versus those who did not to evaluate whether appendicitis manifested as an early sign of malignancy [2]. There was a 4.6 fold increased risk of subsequent cancer after appendectomy, mostly of the digestive organs followed by genitourinary and hematopoietic system, with the risk being highest in the first 3 months. Another study looked at this risk specifically in children [15]. This study showed no increased risk of overall cancer but did show an increased risk of Non-Hodgkin's lymphoma and stomach cancers in those who underwent appendectomy, usually around 15 years after surgery.

To the best of our knowledge, this is the first case report of ALL diagnosed from a pediatric patient who presented and was treated for acute uncomplicated appendicitis. The patient did have a history of intermittent fevers, but there were no other symptoms consistent with leukemia to initiate a further work up. Therefore, this was a very early presentation of ALL manifesting as acute appendicitis.

It is well-known that neutropenic patients with intra-abdominal infections are likely to present with a blunted clinical presentation and fewer symptoms of a lesser magnitude due to a decreased immune response [16,17]. These atypical clinical presentations may result in a delay of diagnosis, which increases the risk of serious complications including perforation, abscess formation, peritonitis, sepsis, and death, particularly if the hematologic disorder has not been yet diagnosed or recognized, such as in our patient [8]. This case report highlights the importance of histopathological evaluation of surgical specimens to rule out malignancy of all kinds, particularly in the setting of abnormal hematologic studies in the pediatric population.

## 4 Conclusion

We present a unique case of B-cell acute lymphoblastic leukemia discovered on pathologic examination of the appendix after laparoscopic appendectomy for acute appendicitis in a 2-year-old female. Clinicians should be aware that, though exceedingly rare, acute appendicitis may be the initial manifestation of underlying leukemia and should be considered in the differential diagnosis of acute appendicitis. If suspected, histopathological evaluation of the appendix may help in the diagnosis.

## Disclosure statement

The views expressed in this manuscript are those of the author(s) and do not reflect the official policy or position of the Department of the Army, Department of Defense, or the US Government.

## Source of funding

This research did not receive any specific grant from funding agencies in the public, commercial, or not-for-profit sectors.

## Ethics approval

This case report was submitted to the Hawaii Pacific Health Research Institute on 3/2/21 and was determined exempt from Institutional Review Board ethical approval. The reference number/HPHRI Study Number for this case is: 2021-021.

## Consent

Written informed consent was obtained form from the patient for publication of this case report and accompanying images. A copy of the written consent is available for review by the Editor-in-Chief of this journal on request.

## Author contribution

Scott R. Marison Jr., MD: Writing – Original Draft, Writing – Review & Editing.

Brooke Pati, MD: Investigation.

Nicole R. Laferriere, MD: Writing – Review & Editing.

Ally Ha, MD, MS: Conceptualization.

Russell K. Woo, MD: Conceptualization, Supervision.

## Research registration

Not applicable.

## Guarantor

Dr. Russell Woo and Dr. Scott Marison.

## Provenance and peer review

Not commissioned, externally peer-reviewed.

## Declaration of competing interest

None.
